# Advances on Microsupercapacitors: Real Fast Miniaturized Devices toward Technological Dreams for Powering Embedded Electronics?

**DOI:** 10.1021/acsomega.2c07549

**Published:** 2023-03-02

**Authors:** Khac Huy Dinh, Pascal Roussel, Christophe Lethien

**Affiliations:** † Institut d’Electronique, de Microélectronique et de Nanotechnologies, Université de Lille, CNRS, Université Polytechnique Hauts-de-France, UMR 8520 - IEMN, F-59000 Lille, France; ‡ Unité de Catalyse et de Chimie du Solide (UCCS), Université de Lille, CNRS, Centrale Lille, Université d’Artois, UMR 8181 − UCCS, F-59000 Lille, France; § Réseau sur le Stockage Electrochimique de l’Energie (RS2E), CNRS FR 3459, 33 rue Saint Leu, 80039 Amiens Cedex, France; ∥ Institut Universitaire de France (IUF), 75005 Paris, France

## Abstract

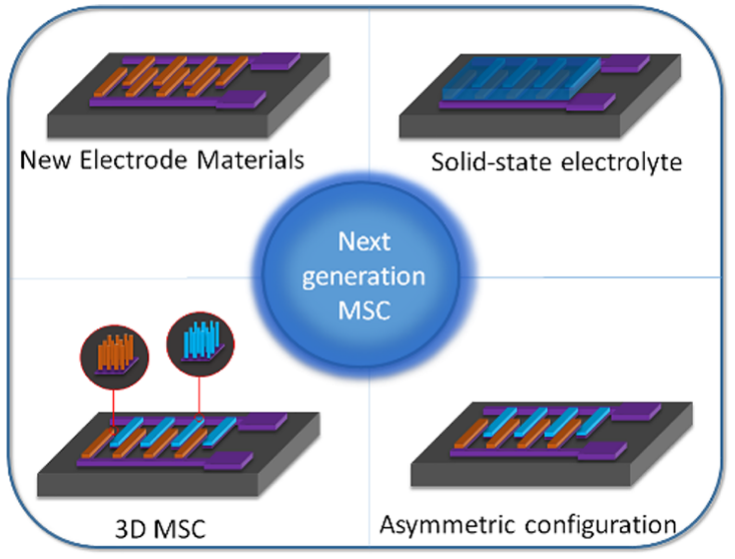

Microsupercapacitors (MSCs) have emerged as the next generation of electrochemical energy storage sources for powering miniaturized embedded electronic and Internet of Things devices. Despite many advantages such as high-power density, long cycle life, fast charge/discharge rate, and moderate energy density, MSCs are not at the industrial level in 2022, while the first MSC was published more than 20 years ago. MSC performance is strongly correlated to electrode material, device configuration, and the used electrolyte. There are therefore many questions and scientific/technological locks to be overcome in order to raise the technological readiness level of this technology to an industrial stage: the type of electrode material, device topology/configuration, and use of a solid electrolyte with high ionic conductivity and photopatternable capabilities are key parameters that we have to optimize in order to fulfill the requirements. Carbon-based, pseudocapacitive materials such as transition metal oxide, transition metal nitride, and MXene used in symmetric or asymmetric configurations are extensively investigated. In this Review, the current progress toward the fabrication of MSCs is summarized. Challenges and prospectives to improve the performance of MSCs are discussed.

## 1 Introduction

**Figure 1:**
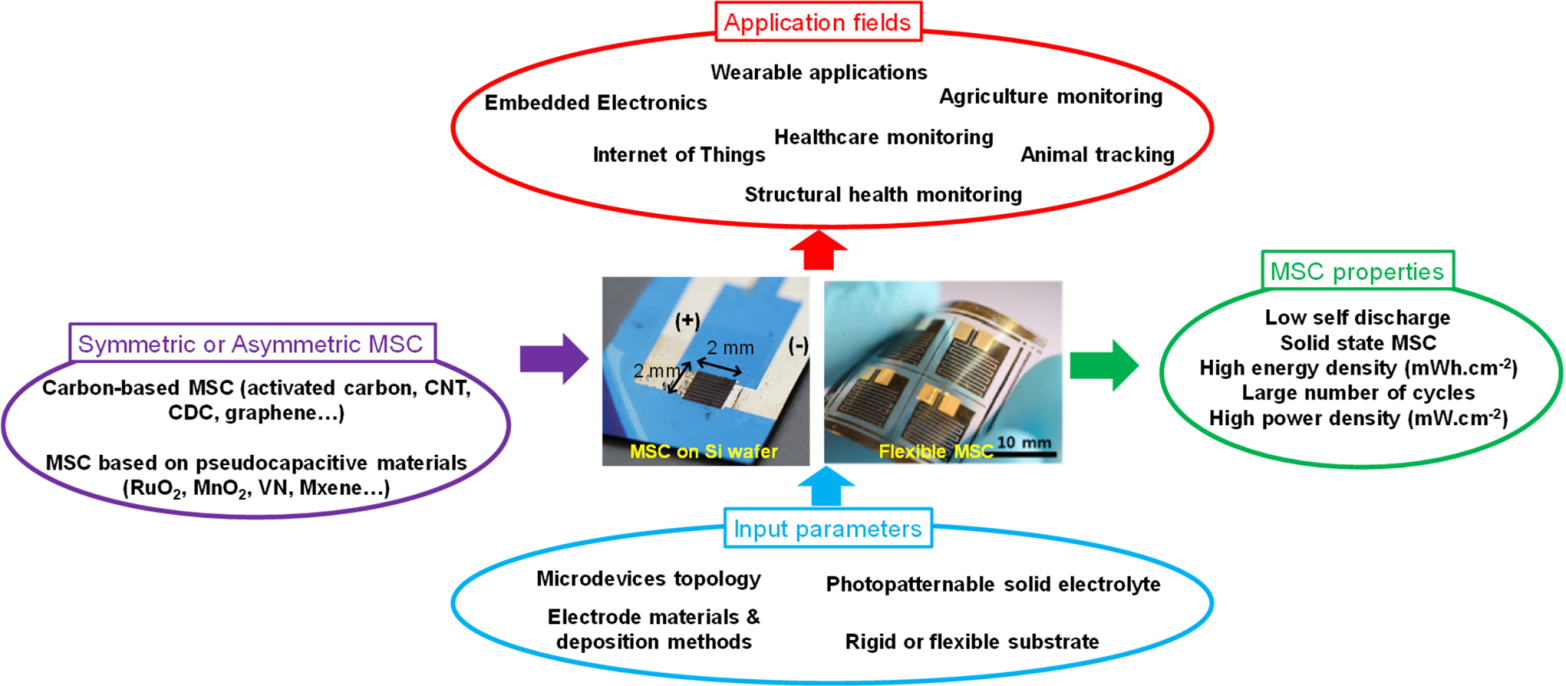
Overview of the MSCs parameters, configuration, properties, and application fields. Reprinted with permission from ref (4). Copyright 2015, American Chemical Society.

Nowadays, the Internet of Things (IoT) is widely developed in our daily life and has numerous advantages (i.e., autonomy; easy access; speedy operation for smart devices, health, and agriculture monitoring).^1,2^ IoT refers to the global network of interconnected devices through the Internet combining wireline and wireless connections to share and exchange data. The application fields are various such as healthcare, industry, agriculture monitoring, and transportation (Figure 1), but the energy dependence is critical at the dawn of a climatic crisis.^3−5^ To get such autonomous and maintenance-free IoT devices, energy storage is intensively integrated as the main power source, but primary cell replacement or recharging of electrochemical energy sources is an issue. Among different kinds of energy storage devices such as conventional capacitors or batteries, electrochemical capacitors and supercapacitor technology^6,7^ are good candidates for fast rate application because of their high power densities, high rate capabilities. and long-life, With widespread system on a chip applications, where many components (sensors,^8^ data management systems,^9^ radio frequency transceivers,^10^ energy sources^11,12^) are integrated on a millimeter chip for healthcare treatment in the body, eyes, or heart with minimum incision, miniaturized devices with a small footprint surface are thus necessary. Microdevices need small energy storage systems to be autonomous.^13,14^ Batteries and electrochemical capacitors are the most comment energy storage system used. However, electrochemical capacitors have high power density and a fast charge–discharge rate but lack energy density compared to batteries.^15^ Therefore, electrochemical capacitor technology has to be downsized to at least millimeter or, better, micrometer scale, leading to a new class of miniaturized devices called microsupercapacitors (MSCs).^1,16,17^ Typically, a MSC is fabricated on a rigid (e.g., silicon)^16,18^ or a flexible substrate (e.g., kapton)^19−21^ depending on the applications. It consists of two current collectors, two electrode materials made from thin or thick film technology deposition methods separated by a solid (ideally) electrolyte with high ionic conductivity. The key point for an MSC is related to its footprint surface, which has to be limited, depending on the available size of the power sources within the IoT device. In that case, to fulfill this requirement, the current collectors, the electrode materials, and the solid electrolyte have to be patterned to limit the footprint surface.^22^

Unfortunately, while the first MSC was published more than 20 years ago,^23,24^ MSCs are not yet commercially off the shelf and the technology is still not at an industrial level in 2023. Various scientific and technological locks have to be overcome in order to raise the TRL (technology readiness level) to an industrial stage. This Review will summarize the latest developments in this domain and will give some prospects/guidelines to be followed for the fabrication of the next generation of MSCs.

## 2 Device Topology

**Figure 2:**
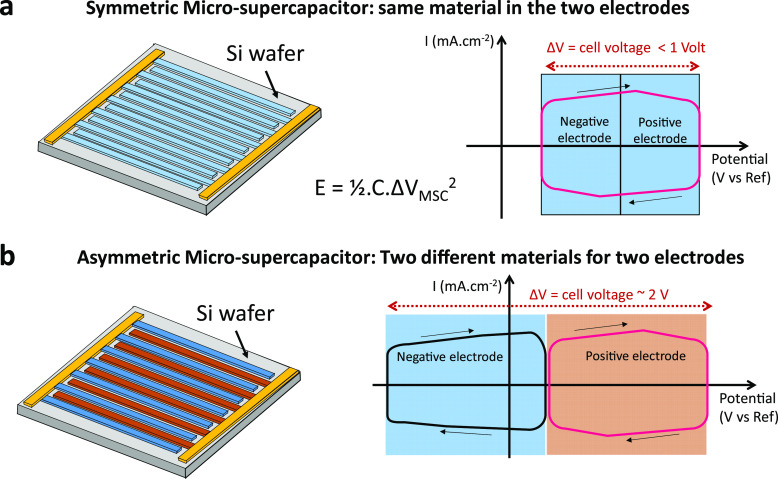
MSC based on an interdigitated topology with a symmetric configuration (a) or an asymmetric configuration (b).

The performance of MSCs is strongly correlated to the device configuration. For conventional electrochemical capacitors (EC), the most common configuration is the parallel-plate one. In that setting, the two electrodes are contacted (sandwiched) through a separator soaked in a liquid electrolyte.^7^ This configuration is thus widely used in EC but rarely selected for MSC because (*i*) a separator could not be easily downsized to millimeter or micrometer scale and (*ii*) the alignment of two different substrates integrating each electrode material is also a difficult task. The parallel plate configuration, or sandwich structure, is nevertheless an efficient topology. However, the large volume occupied by the MSC is the main limitation where the volume is constrained within a miniaturized device. From technological and miniaturization point of views, it is easier to move from a parallel plate configuration to an interdigitated topology^1^ (Figure 2).

It consists of two electrodes made of numerous interpenetrated fingers with no electrical connection between them. The charge storage mechanism arises from ion conduction between the two interdigitated electrodes.^25^

Due to the electrical connection in the same plane, this configuration is ideal for an on-chip system where the surface is generally limited. As described by its name, this setup mostly contains electrodes deposited in the same plane and separated by a blank gap, as depicted in Figure 2b. Due to the gap between the two electrodes, the amount of active material is reduced regarding the footprint surface of the devices, resulting in the decrease in capacitance. Consequently, it is mandatory to reduce the gap between the two electrodes. The thickness of MSC is controlled approximately by the thickness of the electrode.

Besides the topology itself, depending on the choice of the different electrode materials—made from film deposition methods—different classes of MSCs can be elaborated:

• When the electrodes are composed of the same (pseudo)capacitive material, the miniaturized device is called a symmetric MSC (Figure 2a). Consequently, the cell voltage (Δ*V*) of the MSC is limited to the electrochemical window stability of the chosen electrode material, and only half of this electrochemical window is used during the charge/discharge cycles.

• When the electrodes are composed of two different (pseudo)capacitive materials, the MSC is then considered an asymmetric one (Figure 2b). As a result, the MSC benefits from the complementary working potential of the two electrodes, thus improving the cell voltage.

• When the electrodes are composed of a (pseudo)capacitive material combined with a Faradic material, the microdevice does not fall into a class of MSC but is considered a hybrid microdevice, where two different charge storage processes occur within the two electrodes. Even if this class of microdevices is of interest, it is out of the scope of this Review.

## 3 Device Performance: from Macro- to Microscale Electrochemical Capacitors

The fabrication techniques are an important aspect to be considered to improve MSC performance. They are well summarized in the literature^26,27^ and can be listed as photolithography,^28^ femtosecond laser scribbling,^29^ focused ion beam etching,^30^ and inkjet printing.^31^ Each technique has its advantages and drawbacks. For example, photolithography is a low cost lithography method (as compared to e-beam lithography), but a maskless technique is cheaper yet less precise than the photolithography method to pattern the electrode material. In contrast, laser scribbling and ion beam etching are defect-controllable techniques but limited to large-scale production. For commercialization purposes, improving fabrication techniques is necessary to meet the requirement of mass production with high-resolution microdevices while remaining cost-effective.

In electrochemical capacitors, the device performances (capacitance, energy, and power) are reported in F g^–1^, Wh kg^–1^, and W kg^–1^, taking into account electrode materials with a high mass loading (>10 mg cm^–2^) and thickness (>100 μm). These metrics are meaningless for miniaturized electrochemical capacitors where the footprint surface is limited and, thus, the key parameter: in that context, the capacitance, energy, and power densities are preferably reported in F cm^–2^, mWh cm^–2^, and mW cm^–2^, respectively; these metrics are relevant for MSCs and reflect a pertinent overview of the MSC performance. The thickness of the electrode (<50 μm) is significantly lower than that of the bulk electrode of electrochemical capacitors.

Carbon-based MSCs are the first class of MSCs operating in an organic electrolyte or ionic liquid where the charge storage mechanism arises from ion electrosorption in porous carbon electrodes: the performance of MSCs can be maximized if the pore diameter of the carbon electrode matches the ion size of the electrolyte.^32^ Activated carbon,^18^ carbon nanotubes,^33^ carbon-derived carbide,^34,35^ graphene,^19^ MXene,^36−38^ and other allotropes of the carbon are classically investigated as potential electrode materials for MSCs. The cell voltage of this class of MSCs is classically close to 3 V.

The second class of MSCs is based on pseudocapacitive materials operating in an aqueous electrolyte (Δ*V* ∼ 1 V). The charge storage process arises from a fast redox reaction occurring at the surface or subsurface of transition metal oxides^39−42^ (MnO_2_, RuO_2_, ...) or transition metal nitrides^22,28,43−48^ (VN, WN, MoN, ...). The capacitance values issued from pseudocapacitive materials (i.e., from a redox process) are significantly higher than those of carbon materials.

However, until now, the main drawback of the MSCs is their relatively low energy density, due to the low amount of active materials, related to the low thickness of the electrode materials. Many attempts have been made to improve the performance of MSC. Since the energy density (in mWh cm^–2^) of a MSC is given by *E* = 1/2*C*_s_ × Δ*V*^2^, where *C*_s_ is the surface capacitance (in mF cm^–2^) and Δ*V* is the cell voltage (in volts), the schematic strategy consists, thus, of increasing either (both) the capacitance value *C*_s_ (surface or areal capacitance) or (and) the cell voltage Δ*V* playing with the electrode material’s type or (and) the device topology (parallel plate, interdigitated, symmetric, or asymmetric, see Figure 2). The performance of MSCs is not only related to its energy density, and in fact, it is evaluated by many features, and an ideal MSC should combine numerous properties such as a long life cycle, high rate capabilities, environmental friendliness, and a low self-discharge rate, besides the already evoked high energy and power densities. Coulombic efficiency, which is the ratio of discharge to charge capacity, can give an idea about the cycle life and rate capabilities of MSCs.^49^ Depending on electrode material, Coulombic efficiency can vary from 97% for the amorphous TiO_2_ Electrode^50^ to 100% (Fe,Mn)_3_O_4_ for spinel oxide.^51^

Recently, alternate current (AC) line-filtering has been one of the new important aspects to consider in the performance of MSCs, opening new avenues for commercializing applications. The fundamental mechanism relies on the frequency response, particularly at 120 Hz, characterized by Nyquist and Bode plots of MSCs.^52^ Since 2010, graphene double-layer capacitor reports show that AC line filtering properties lead to new ways of replacement of bulky electrolytic capacitors, especially for IoT application.^53^ Following this, a conducting polymer^54^ and MXene^55^ were also applied to AC line filtering. However, the trade-off between capacitance and frequency is a problem to overcome because MSCs are energy storage devices in general: a large capacitance value leads to a high time constant (τ = RC), giving rise to a limitation in the frequency domain for AC line filtering.

Last but not least, one of the main limitations of the MSCs deals with the development of all solid-state technology allowing a high rate capability (with solid electrolyte showing high ionic conductivity) while keeping the energy density at the highest level.

**Figure 3:**
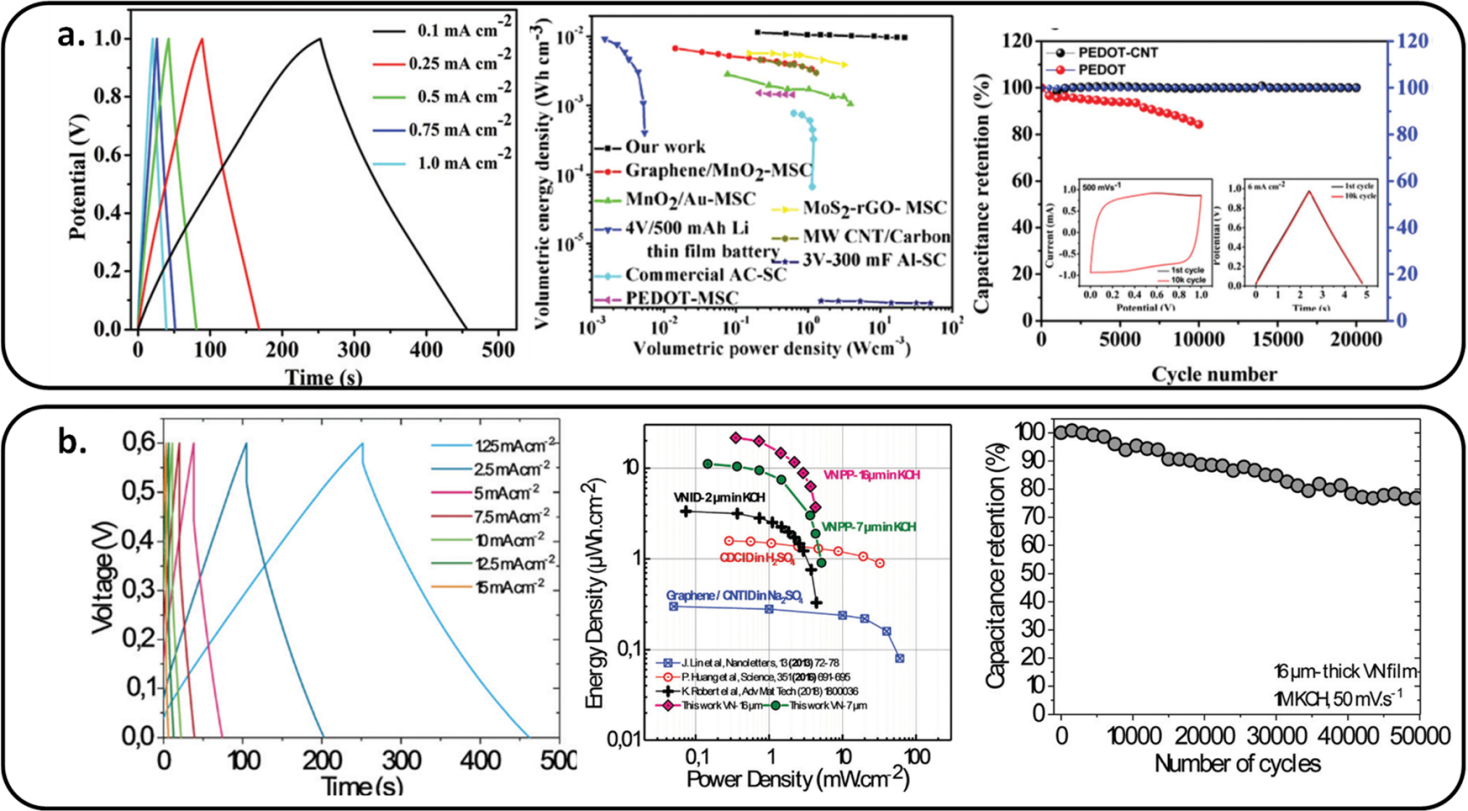
Performance of MSC based on (a) nitrogen-doped graphene film. Reprinted with permission from ref (56). Copyright 2019, Royal Society of Chemistry. (b) Vanadium nitride film. Reprinted with permission from ref (45). Copyright 2020, Royal Society of Chemistry.

Depending on the nature of the material electrode and electrolyte, MSCs have to sacrifice one or some of those features. For example, the nitrogen-doped graphene film electrode showed 90.1% capacitance retention after 100 000 cycles with a high mass loading of 11.2 mg cm^–2^, but the capacitance stayed low at 20.6 mF cm^–2^ in an aqueous electrolyte^56^ (Figure 3a). In contrast, a VN film electrode can archived up to 1.2 F cm^–2^ in an aqueous electrolyte, but capacitance retention is about 80% after 50 000 cycles for a 16-μm-thick film^45^ (Figure 3b). Specifically, a lower energy density than the practical requirement is a challenge that limits the application of MSCs. A recent paper published in 2023 shows a significant improvement of the cycling and aging performance of sputtered vanadium nitride films with no loss of the initial capacitance value after 150 000 cycles and no degradation of the electrode performance after 13 months.^57^

Up to now, there have been no commercially available MSCs delivering sufficient performance for powering miniaturized IoT devices. There are some issues both at the electrode material level and at the electrolyte level. Various “classical” electrode materials such as carbon-based, transition metal oxide, or nitride and conductive polymers are currently integrated within a MSC. Nevertheless, new materials are promising. Among others, we can cite MXenes and ternary materials in a multicationic or multianionic configuration in order to boost the performance of such microdevices.

## 4 Overview of the Most Explored (Pseudo)capacitive Electrode Materials for MSC

MSCs can be divided into two main classes based on the storage charge mechanism, namely, electrical double layer capacitors (EDLC) and pseudocapacitors.^10^ The EDLC stores charges via an electrostatic charge absorption/desorption mechanism at the electrode/electrolyte interface.^58^ The most common material for such an MSC is porous carbonm where it is important to match the pore size (from the electrode materials) with the ion size (from the electrolyte) to maximize the storage capabilities.^16,32^

On the other hand, pseudocapacitors store charges via fast redox reactions at the surface or subsurface of the active material without phase transformation of the electrode material.^59^ Conductive polymers and transition metal oxide or nitride are well-known materials for such pseudocapacitors. 2D MXene electrodes are challenging materials to be included within a pilot production line of MSCs based on the vacuum deposition technique. The charge storage process of the main transition metal oxide and nitride material was unveiled by various groups based on numerous *in situ*/*operando* techniques.

For example, the charge storage mechanism of transition metal oxide material RuO_2_ upon protonation^60^ is shown in eq 1:
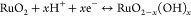
1where 0 < *x* < 2.

Among all of the pseudocapacitive materials, MnO_2_ is an earth-abundant, environmentally friendly, low-cost oxide and exhibits a pseudocapacitive behavior in neutral aqueous electrolytes despite a low electronic conductivity. The charge storage process in MnO_2_ consists of the fast intercalation of protons (H^+^) and/or cations (C^+^ = Na^+^, Li^+^, K^+^, ...) coming from the aqueous electrolyte at the surface or near the surface of the MnO_2_ such as that described^39,40^ in eq 2:
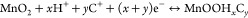
2where C^+^ = Na^+^, Li^+^, K^+^, etc. and *x* and *y* correspond to the number of moles of H^+^ and C^+^ intercalated in MnO_2_.

The charge mechanism of transition metal nitride such as vanadium nitride (VN) is given in eq 3, where takes place from OH^–^ in formation of the double layer and fast redox reactions on the surface of VN_*x*_O_*y*_:
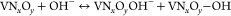
3The hydroxyl ion (OH^–^) is involved in the formation of the electrical double layer as well as in the fast faradic redox reaction occurring on the surface of the partially oxidized vanadium nitride.^44^ While nanostructured VN particles were classically used as an electrode material for macroscale electrochemical capacitors, a question arises about the electrochemical behavior of VN films for MSC. Consequently, the charge storage mechanism of sputtered VN films for MSC was unveiled recently in 1 M KOH by combining *in situ* atomic force microscopy techniques, transmission electron microscopy, and operando X-ray absorption spectroscopy measurement. This set of analyses revealed the presence of V^3+^ and V^4+^ elements within the sputtered VN films. More specifically, it clearly evidenced a change in oxidation state from V^3+^ to V^4+^ upon electrochemical oxidation of the films in 1 M KOH.

Finally, since the introduction of MXene in 2011 from the Barsoum and Gogosti groups (Drexel University, Philadelphia, PA), two-dimensional transition metal carbide or nitride materials have been investigated as pseudocapacitive electrodes for both electrochemical capacitors and MSCs.^36,61^ Investigation of the charge storage process of the most studied Ti_3_C_2_ MXene material reveals that the surface chemistry of this 2D material plays an important role depending on the used synthesis process to transform MAX phase materials to a 2D MXene electrode. The electrochemical properties of MXene are widely correlated to the insertion behavior on cations (coming from the electrolyte) between the sheets. In a neutral or basic electrolyte, a large number of solvated cations (Li^+^, Na^+^, K^+^...) with numerous sizes can be inserted in between the MXene layers.^62^ In acidic solutions such as 1 M H_2_SO_4_, oxygen terminations of the Ti_3_C_2_ “clay electrode” with the protons coming from the acidic electrolyte induce fast redox reactions revealing a pseudocapacitive behavior.^36^ Finally, in a nonaqueous electrolyte such as the 1 M LiPF_6_/EC/DMC electrolyte, Ti_3_C_2_ MXene synthesized via the Lewis acid molten salt method shows a fast intercalation pseudocapacitance behavior attributed to the insertion of a lithium cation between the MXene layers in a large potential window.^63^

Porous carbons are the most popular electrode materials used as an efficient electrode for MSC; the main issue consists of depositing/growing the carbon electrode as a film with limiting the footprint surface on a substrate.^64^ The charge storage mechanism (electrosorption process) in the porous carbon is similar to that of bulk electrodes for electrochemical capacitors and is known as electrical double layer capacitance (EDLC).^10^ Therefore, the main challenge is to keep the large surface area of the porous carbon and to match the pore size of this material with the ion size coming from the electrolyte.

**Figure 4:**
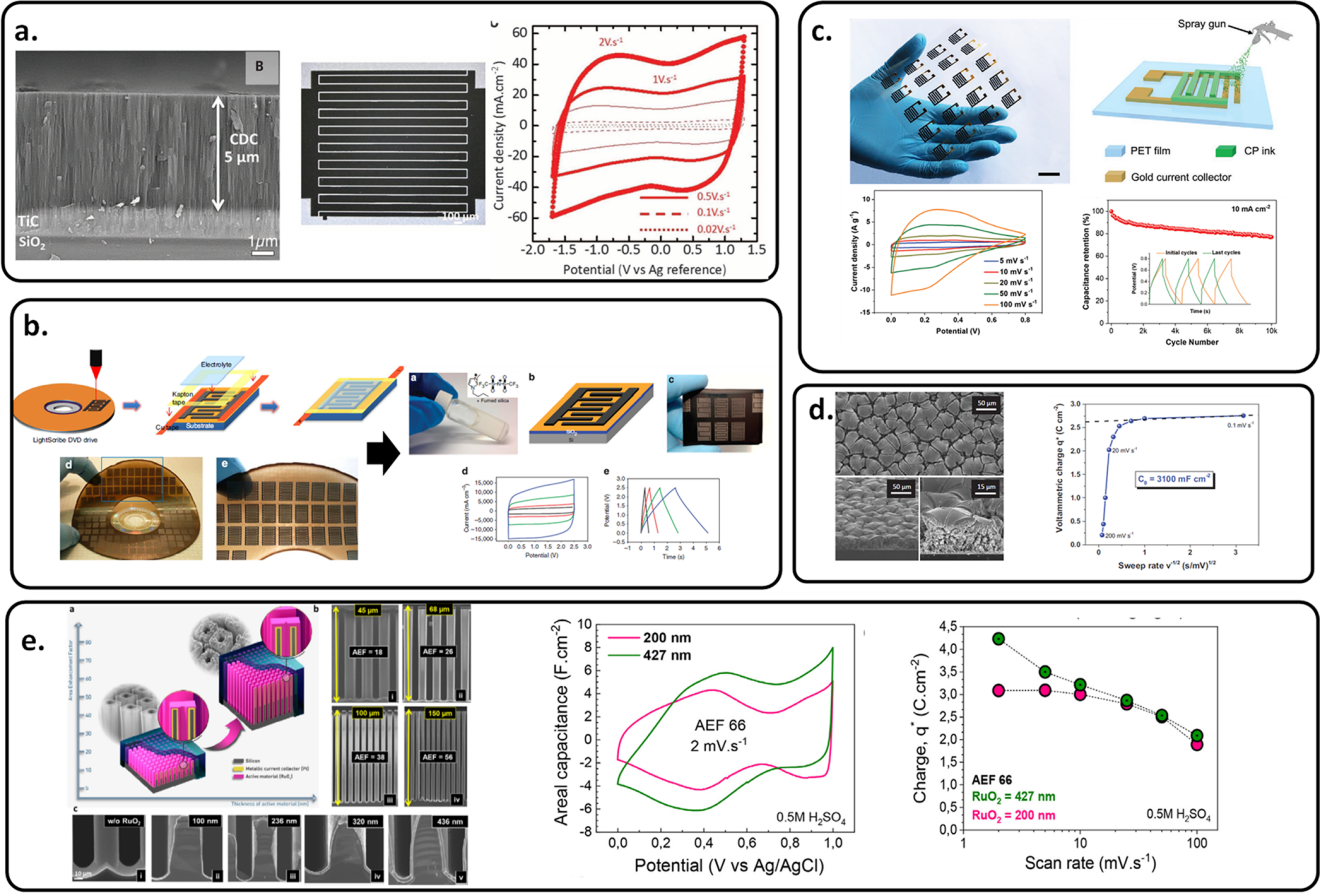
Overview of the electrode material used in MSC. (a) Carbide-derived carbon on a Si wafer. Reprinted with permission from ref (16). Copyright 2016, American Association for the Advancement of Science. (b) laser-scribed graphene MSC. Reprinted with permission from ref (19). Copyright 2013, Springer Nature. (c) PANI. Reprinted with permission from ref (71). Copyright 2021, John Wiley and Sons. (d) RuO_2_ film on 3D porous Au. Reprinted with permission from ref (72). Copyright 2015, John Wiley and Sons. (e) RuO_2_ film on 3D scaffold. Reprinted with permission from ref (73). Copyright 2021, Elsevier.

In 2010, carbon-based MSCs were proposed^18^ using either carbon onions (onion-like carbon, OLC) or activated carbon (AC) as the active electrode material. The carbon nanoparticles were deposited from a colloidal suspension using an electrophoretic deposition technique (EPD) on interdigitated Ti/Au current collectors made on a Si wafer. The OLCs are interesting for high power applications, and the MSC made from OLC reported a remarkable rate performance at 200 V s^–1^. MSCs based on AC exhibited one of the highest energy densities (20 μWh cm^–2^) reported so far (in 2010) in an organic electrolyte (3 V). The collective fabrication of MSCs based on a carbide-derived carbon (CDC) thin film was proposed in 2016. Microfabrication techniques were used to deposit, pattern, and etch sputtered TiC film. After a chlorination process able to convert TiC into TiC-CDC, the as-fabricated MSC on a Si wafer delivered both high areal energy (30 μWh cm^–2^) and high power density (30 mW cm^–2^) in porous carbon nanosheets doped with rich nitrogen, which are reported to deliver a high energy density of 8.4 mWh cm^–3^ at a power density of 24.9 mW cm^–3^.^65^ Using a controllable activation method, a 3D bicontinuous porous carbon MSC is fabricated with an area energy density of 4.9 μWh cm^–2^ and a volume energy density of 11.13 mWh cm^–3^.^66^ MSCs based on N/O codoped graphene quantum dots showed an energy density of 1.4 μWh cm^–2^.^67^ In 2013, M. F. El-Kady et al. used a commercial LightScribe DVD burner process to make a laser-scribed graphene MSC (collective fabrication) where more than 100 MSCs were fabricated on a kapton film having the same diameter as a DVD disc, as shown in Figure 4b, and delivered a total power density of 200 W cm^–3^.^19^ Li et al. reported SiC@C nanowire arrays delivering an energy density of 2.84 μWh cm^–2^ at a power density of 65.1 μW cm^–2^, which is 700% higher than that of a pure SiC electrode, benefiting from a large cell voltage of 2.6 V.^68^ Conductive polymers are also promising candidates for wearable MSCs due to their flexible and printable abilities, but they suffer from a low ionic transfer, and thus low cycling stability and rate capability. To tackle the low cycling stability, Tahir et al., in 2020, grew polypperol (PPy) in reduced graphene oxide (rGO) on micropatterned Au, which achieved 82% capacitance retention after 10 000 cycles in a 2 M KCl electrolyte and the so-fabricated MSC delivered 4.3 μWh cm^–2^ energy density at 0.36 W cm^–2^ power density.^69^ In 2021, Chu et al. used polyaniline (PANI) ink with conductive carboxylic multiwalled carbon nanotube (C-MWCNT) networks to increase the rate capability of the PANI electrode by 73.7%. The PANI ink can be printed on different substrates to form flexible MSCs and deliver an energy density of 2.6 mWh cm^–3^ and 84.6% capacitance retention after 1000 bending cycles.^70^ Additionally, PANI with no additive is stable in air, where a 96.6 mF cm^–2^ areal capacitance has been reported by Chu et al. (Figure 4c). The MSCs based on PANI/CA (citric acid) nanosheets delivered an energy density of 2.4 mWh cm^–3^ at a power density of 238.3 mW cm^–3^.^71^

Recently, pseudocapacitive materials such as transition metal oxide or nitride have emerged as efficient material electrodes for MSCs. Robert et al. reported a 16-μm-thick vanadium nitride (VN) film deposited by magnetron sputtering delivering a surface capacitance of 1.2 F cm^–2^ and a 25 mWh cm^–2^ of energy density for the full MSC.^45^ Ferris et al. fabricated 3D MSCs by electrodeposition of RuO_2_ material on porous Au, achieving a capacitance value of 3 F cm^–2^ (Figure 4d).^72^

Using the same RuO_2_ material, but on a 3D scaffold made using the deep reactive ion etching method on a silicon wafer, Asbani et al. reported in 2021 a significant increased surface capacitance value of 4.5 F cm^–2^ at 2 mV s^–1^. Besides a high areal capacitance, one additional advantage of this design compared to the previous one on porous metal is its ability to work at a high scan rate, keeping for instance more than 50% of the initial capacitance at 100 mV s^–1^, as depicted in Figure 4e.^73^ Additionally, Bounor et al. reported a 3D MSC based on pulsed electrodeposition^74,75^ MnO_2_ delivers energy densities in a range of 0.05–0.1 mWh cm^–2^ at a power density > 1 mW cm^–2^.^76^ The MSC performances are reported in Table 1.

**Table 1:** Electrochemical Performance of MSCs on Topology and Electrode Material

electrode material	electrolyte	device topology	cell voltage	specific energy	specific power	ref
AC	1 M Et_4_NBF_4_/anhydrous propylene carbonate	symmetric	0–3 V	20 μWh cm^–2^	80 mW cm^–2^	(16)
TiC-CDC	2 M EMIBF_4_ in AN	symmetric	0–0.9 V	30 μWh cm^–2^	30 mW cm^–2^	(5)
MnO_2_/PEDOT:PSS–rGO@CF	Na_2_SO_4_–CMC	asymmetric	0–2.8 V	295 μWh cm^–2^	14 mW cm^–2^	(77)
porous carbon	EMIMBF_4_	symmetric	0–4 V	8.4 mWh cm^–3^	24.9 mW cm^–3^	(65)
porous carbon	LiTFSi	symmetric	0–2.5 V	1.53 μWh cm^–2^	7.92 mWh cm^–2^	(66)
graphene	PVA/H_3_PO_4_	symmetric	0–1 V	1.4 μWh cm^–2^	25 mW cm^–2^	(67)
graphene	PVA/LiOH	symmetric	0–1 V	51.2 μWh cm^–2^	0.968 mW cm^–2^	(31)
G-CNT	PVA/H_3_PO_4_	symmetric	0–1 V	1.36 μWh cm^–2^	0.25 mW cm^–2^	(78)
SWCNT	PVA/H_3_PO_4_	symmetric	0–0.8 V	1 μWh cm^–2^	20 μW cm^–2^	(79)
carbon nanowire	EMIMNTf_2_	symmetric	0–2.6 V	2.84 μWh cm^–2^	65.1 μW cm^–2^	(68)
polypperol//PEDOT	2 M KCl	asymmetric	0–1.4 V	4.3 μWh cm^–2^	0.36 W cm^–2^	(69)
polyaniline	PVA/H_2_SO_4_	symmetric	0–0.8 V	2.6 mWh cm^–3^	59.5 mW cm^–3^	(70)
polyaniline	PVA/H_2_SO_4_	symmetric	0–0.8 V	2.4 mWh cm^–3^	238.3 mW cm^–3^	(71)
VN	1 M KOH	symmetric	0–0.6 V	25 mWh cm^–2^	4 W cm^–2^	(45)
MnO_2_	5 M LiNO_3_	symmetric	0–1 V	0.05–0.1 mWh cm^–2^	>1 mW cm^–2^	(76)
Ti_3_C_2_T_*x*_	PVA/H_2_SO_4_	symmetric	0–0.5 V	0.32 μWh cm^–2^	11.4 μW cm^–2^	(80)
Ti_3_C_2_T_*x*_	PVA/H_2_SO_4_	symmetric	0–0.6 V	51.7 μWh cm^–2^	5.7 mW cm^–2^	(81)
hydrated RuO_2_	0.5 M H_2_SO_4_	symmetric	0–0.9 V	91 μWh cm^–2^		(82)
AC	EMIM/TFSI	symmetric	0–3 V	463.1 μWh cm^–2^	2.0 mW cm^–2^	(83)
Ti_3_C_2_T_*x*_	1 M H_2_SO_4_	symmetric	0–1.2 V	75.5 mWh cm^–3^	1088 mW cm^–3^	(84)
VN//hRuO_2_	1 M KOH	asymmetric	0–1.15 V	20 μWh cm^–2^	3 mW cm^–2^	(85)
P-TiON//VN	LiCl/PVA	asymmetric	0–1.8 V	32.4 μWh cm^–2^	0.9 mW cm^–2^	(86)
Ti_3_C_2_T_*x*_//polypyrrole/MnO_2_	PVA/H_2_SO_4_	asymmetric	0–1.2 V	6.73 μWh cm^–2^	204 μW cm^–2^	(87)
Ti_3_C_2_T_*x*_//AC	PVA/Na_2_SO_4_	asymmetric	0–1.6 V	3.5 mWh cm^–3^	100 mW cm^–3^	(88)
Ti_3_C_2_T_*x*_//rGO	PVA/H_2_SO_4_	asymmetric	0–1 V	8.6 mWh cm^–3^	0.2 W cm^–3^	(89)

## 5 Emerging Materials for Designing Efficient Electrodes for MSCs

**Figure 5:**
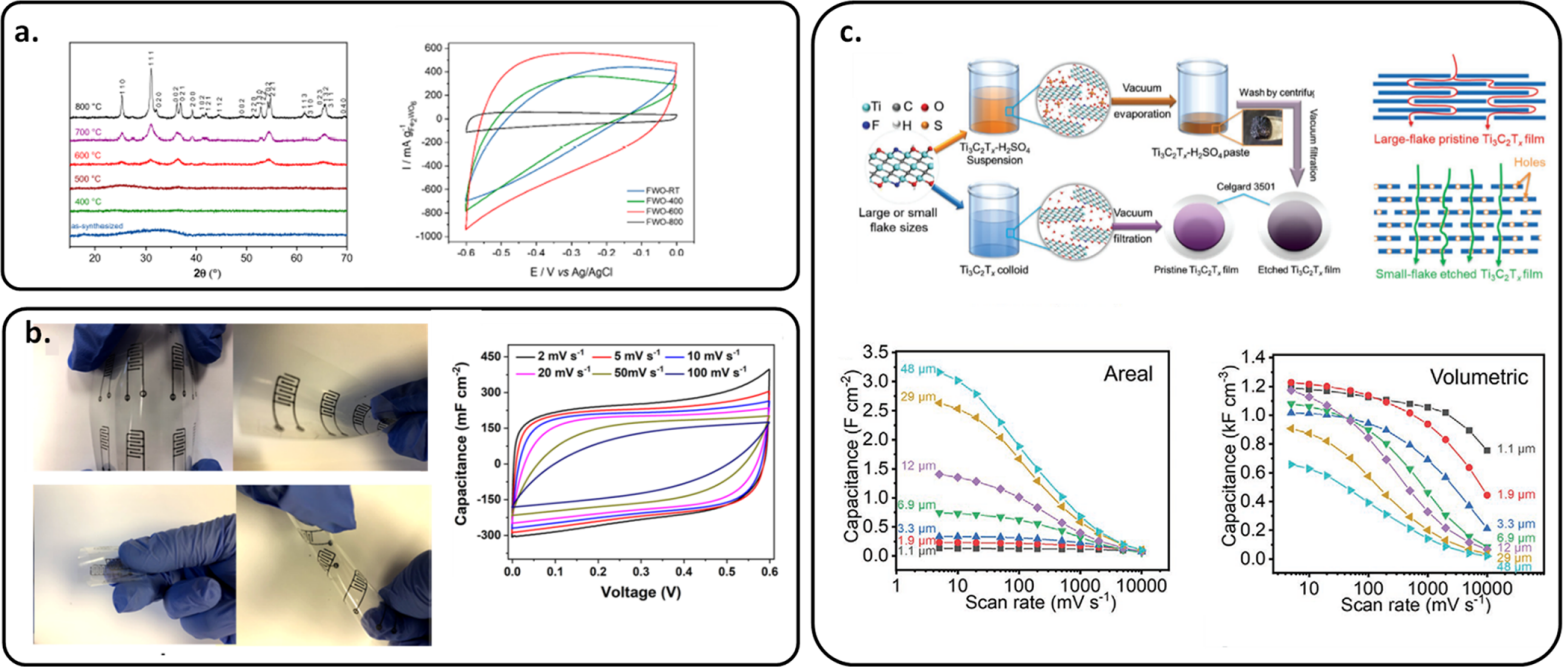
Emerging materials as an efficient electrode for MSCs. (a) Fe_2_WO_6_ material. Reprinted with permission from ref (96). Copyright 2021, Multidisciplinary Digital Publishing Institute. (b) Flexible MSC based on MXene in an aqueous electrolyte. Reprinted with permission from ref (81). Copyright 2020, American Chemical Society. (c) MXene electrode with high surface and volumetric capacitance values. Reprinted with permission from ref (102). Copyright 2021, John Wiley and Sons.

New materials are currently being investigated as efficient electrodes for MSCs. Ternary compounds—that is to say, multicationic^90^ or multianionic^91^ materials—have recently been shown to be good potential candidates to be used in MSCs, taking benefits from the properties of the different elements. High-density multicationic oxides (ternary transition metal oxides) with one transition metal ion and one electrochemically active or inactive metal ion have been widely investigated as an alternative solution. The coexistence of two different cations in a single crystal structure could improve the electrochemical performance as compared to their constituting binary metal oxides.^92−94^ In that context, the group of Brousse has recently demonstrated that FeWO_4_ is an interesting negative electrode for electrochemical capacitors.^92^ More especially, FeWO_4_ was proposed as thin film electrode for MSC using deposition method compatible with semiconductor techniques^95^ to be integrated in a technological process on pilot production line. Besides FeWO_4_, Fe_2_WO_6_ as a negative electrode was synthesized by a polyol-mediated method and showed 240 F cm^–3^ of volumetric capacitance with capacitance retention of 85% after 10 000 cycles (Figure 5a).^96^

Recent research has shown that Mn-based spinels, particularly MnFe_2_O_4_, demonstrate a wide working potential range in an aqueous medium and a pseudocapacitance mechanism close to that of MnO_2_, suggesting favorable properties for both high-energy and power applications.^51,93,94^ Sputtered TiVN ternary films were also investigated as an electrode for MSC and exhibit a surface capacitance value of 15 mF cm^–2^ in KOH aqueous electrolyte.^97^

MXenes,^36,98,99^ the family of early transition metal carbides, nitrides, or carbonitrides, have also been widely investigated as efficient electrodes, due to the high rate/high capacitance properties issued from their two-dimensional properties.^100^ Among MXene materials, Ti_3_C_2_T_*x*_ is the most common candidate with both high conductivity and volumetric capacitance. In 2019, Zhang et al. reported an MXene ink with no additive that can be directly printed to form a MSC with a volumetric capacitance of 562 F cm^–3^ and an energy density of 0.32 μWh cm^–2^.^80^ In 2020, Orangi et al. designed a 3D MSC by direct printing of Ti_3_C_2_T_*x*_ MXenes, layer by layer, with flexible properties due to a polymer substrate, delivering an areal capacitance of 1035 mF cm^–2^ and an energy density of 51.7 μWh cm^–2^ (Figure 5b).^81^ Wu et al. used sodium ascorbate to cap in MXene to improve the oxidation resistance in ambient air for more than 80 days, and MSCs based on this material delivered 108.1 mF cm^–2^ of surface capacitance.^101^ To avoid the restacking problem of MXene, in 2021, Tang et al. used a controllable H_2_SO_4_ oxidation method to optimize the ion pathway (Figure 5c). This method allows the scan rate to raise impressive values up to 10 000 mV s^–1^ and an areal capacitance of ∼3.2 F cm^–2^.^102^

## 6 Development of Solid-State MSCs

In MSCs, as in other electrochemical energy storage systems, the electrolyte plays a crucial role in making the ionic connection between the two electrodes. Therefore, it drastically governs and limits the performance of MSCs such as the maximum operation cell voltage, working temperature range, lifetime and capacitance of the miniaturized devices, among others. The electrolyte can be roughly divided into two categories: liquid and solid/quasi-solid-state electrolytes.^103^ Among the liquid-based electrolytes, aqueous ones have high ionic conductivity but suffer, besides a low operating temperature range, from a restricted voltage window (1.23 V) due to water electrolysis (electrochemical water splitting).^104^ An organic electrolyte is classically used with porous carbon electrodes allowing operation up to 3 V cell voltage.^105^ More importantly, liquid electrolyte is difficult to apply for MSCs due to leakage issues. On the other hand, solid-state electrolytes can provide wide cell voltage with restricted footprint area, making them some good compromise potential candidates for MSCs. However, the main drawback of solid-state electrolytes is their low ionic conductivity. In 2001, Yoon et al. studied LiPON (L_i2.94_PO_2.37_N_0.75_) thin films, acting as a solid electrolyte for RuO_2_ thin film microsupercapacitor, but the low ionic conductivity induced some rate limitation and, thus, low capacity and power density, besides no cycling performance.^23^

To increase the ionic conductivity of solid-state electrolytes, using gel-polymer electrolytes such as hydrogel or ionogel is an attractive solution. This kind of electrolyte consists of polymer network with a solvent trapped inside. Among several polymers (i.e., polyethylene oxide, polymethyl methacrylate, polyvinylidene fluoride), polyvinyl alcohol (PVA) is an attractive candidate due to its nontoxicity, chemical stability, and good mechanical properties.^106^ Depending on the solvent nature (water or ionic liquid), they are called hydrogels or ionogels electrolytes, respectively. Many studies have been reported for hydrogel electrolytes such as PVA/KOH and PVA/H_2_SO_4_.^103^ However, the major drawback of hydrogels is water evaporation, which strongly limits their application for MSCs.

**Figure 6:**
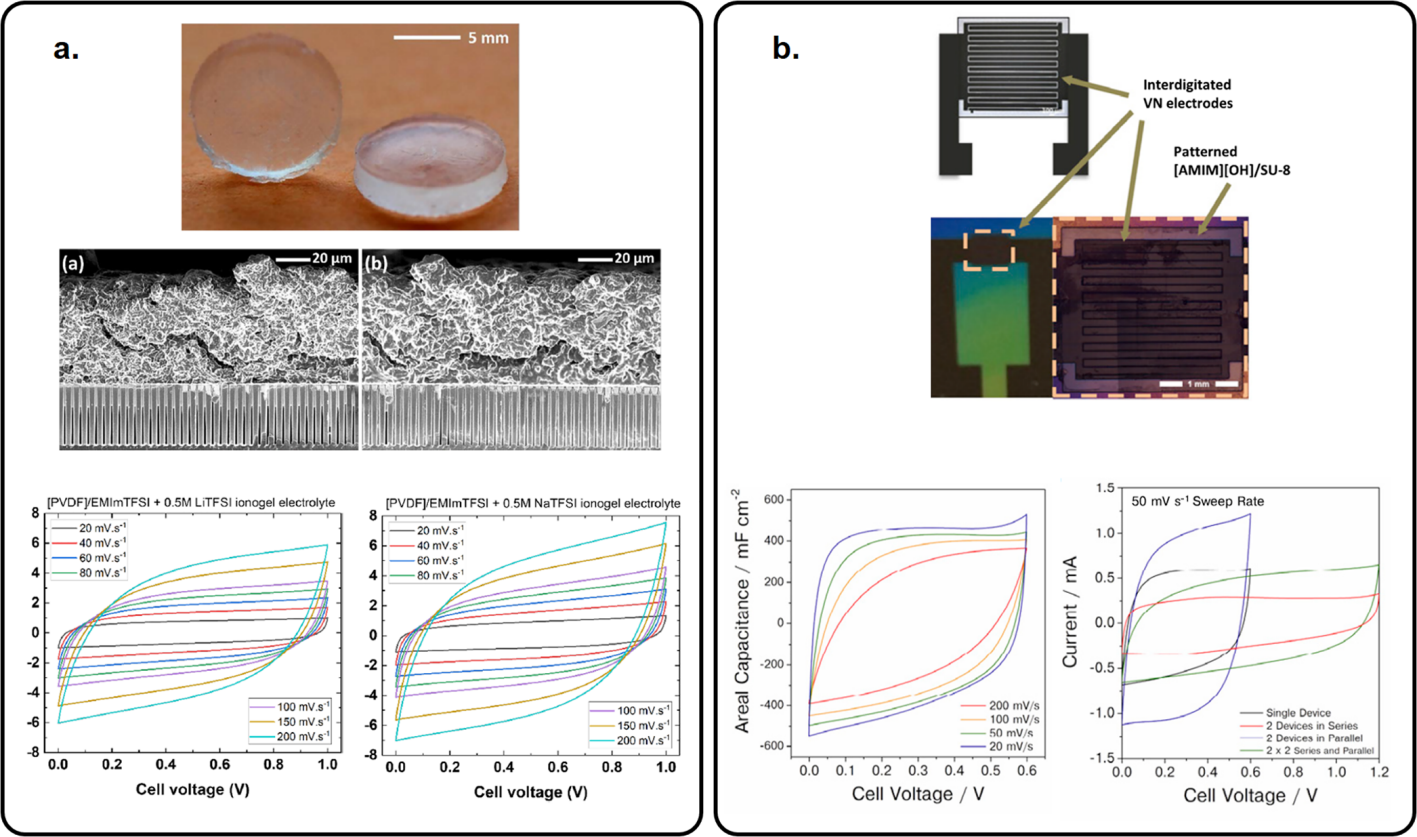
Overview of solid-state MSCs. (a) EMImTFSI-based ionogel electrolyte. Reprinted with permission from ref (5). Copyright 2022, Elsevier. (b) Photopatternable [AMIM][OH]/SU-8 electrolyte for MSC. Reprinted with permission from ref (22). Copyright 2021, Elsevier.

The water evaporation does not take place in ionogel electrolytes, making them an interesting potential candidate for solid state MSCs.^19,107−109^ Both the ionic and electrode material compatibility need however to be considered to maximize MSC performance.^3^ For this purpose, Guillemin et al. added lithium and sodium salts to 1-ethyl-3-methylimidazolium bis(trifluoromethanesulfonyl)imide-based (EMImTFSI) ionogels (Figure 6a).^5^ The conductivity is only slightly decreased, but global capacitive performance is greatly enhanced, and 3D interdigitated MnO_2_/MnO_2_ MSCs have been shown to keep 85% capacitance retention after 50 000 cycles.

For industrial application, it is however mandatory for MSCs to be scaled up, and to restrict the surface of ionogel to several square millimeters is an issue. Choi et al. reported photopatternable technology^110^ to produce solid electrolytes directly on interdigitated MSCs based on vanadium nitride films. SU-8 photoresist was used as a host polymer matrix where 1-allyl-3-methylimidazolium hydroxide ions [AMIM][OH] are trapped within the matrix. Due to the photopatternable capability of SU-8 photoresist, the [AMIM][OH]/SU-8 electrolyte was cast onto VN/VN MSCs to form single devices as well as connected devices (Figure 6b).^22^

## 7 From Symmetric to Asymmetric Configuration to Improve the Cell Voltage

**Figure 7:**
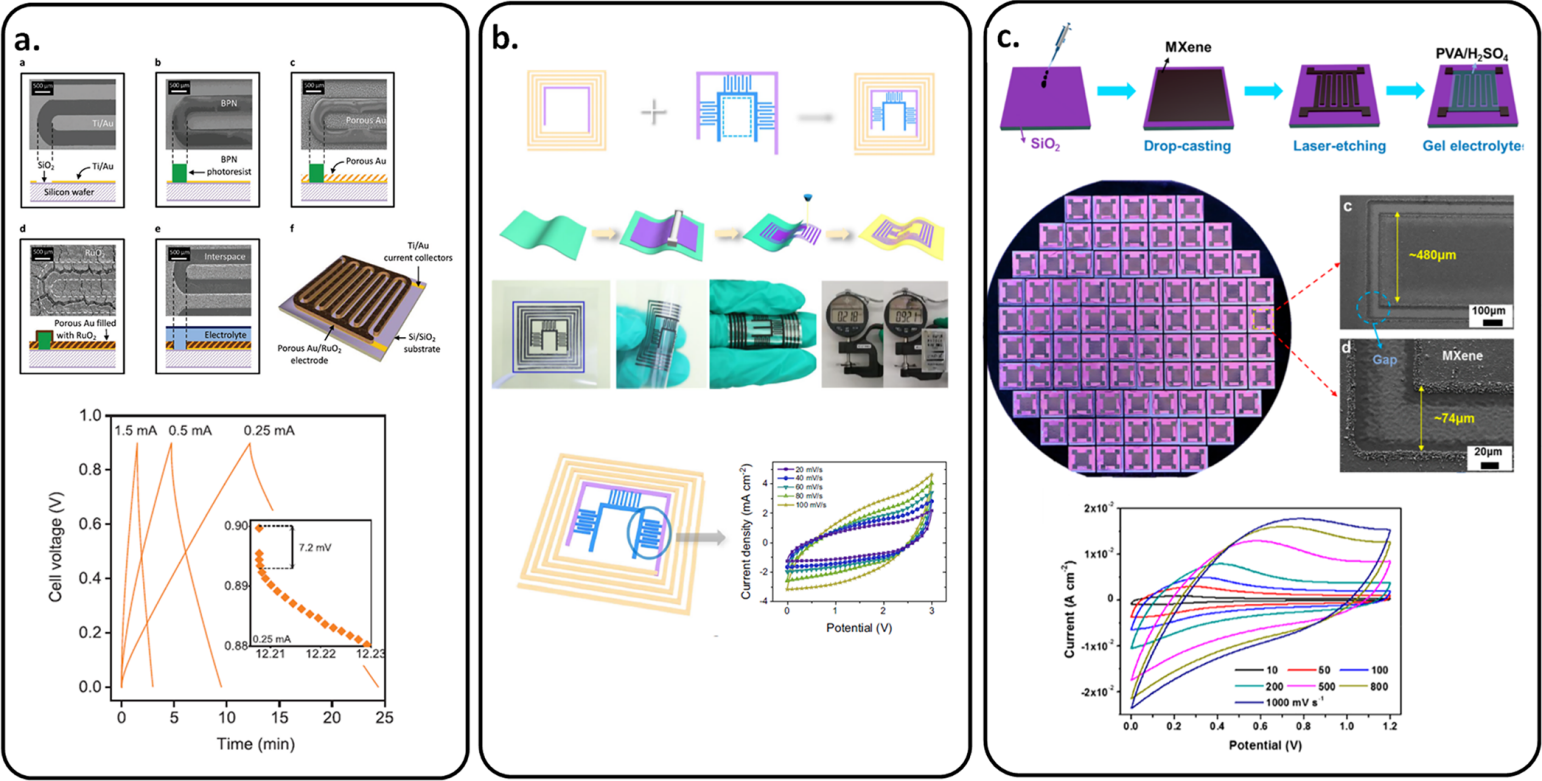
Overview of several symmetric MSCs on rigid or flexible substrates. (a) Symmetric RuO_2_//RuO_2_ MSC. Reprinted with permission from ref (82). Copyright 2019, John Wiley and Sons. (b) Symmetric AC//AC MSC. Reprinted with permission from ref (83). Copyright 2021, Springer Nature. (c) Symmetric MXene//MXene MSC. Reprinted with permission from ref (84). Copyright 2022, American Chemical Society.

As previously mentioned, another approach to strongly increase energy density (square law) of MSCs is to increase the cell voltage. The cell voltage of symmetric MSC (same material for cathode and anode) is limited by the working potential windows of the (pseudo)capacitive materials which is used in both of the two electrodes. For example, Δ*V*_VN_ = 0.6 V in 1 M KOH electrolyte,^45^ Δ*V*_Ti_3_C_2_T*_x_*_ = 0.6 V in PVA/H_2_SO_4_ gel electrolyte,^81^ and Δ*V*_MnO_2__ = 1 V in 5 M aqueous LiNO_3_.^76^ A symmetric MSC based on hydrated RuO_2_ was proposed by Ferris et al. where the pseudocapacitive material was deposited onto gold–copper 3D porous current collectors (Figure 7a). This MSC (Δ*V* = 0.9 V) exhibited a cell capacitance of 812 mF cm^–2^ at an energy density of 329 mJ cm^–2^ (∼91 μWh cm^–2^).^82^ Gao et al. designed a MSC composed of a wireless charging coil and electrode to form a seamlessly integrated wireless charging MSC (Figure 7b). This design gave a capacitance of 454.1 mF cm^–2^, a cell voltage of 3 V, and an energy density of 463.1 μWh cm^–2^, coupled with a contactless charging ability.^83^ Finally, as reported by Huang et al. in 2022, MXene films drop-casted on a SiO_2_ substrate were shown to exhibit a cell voltage Δ*V* = 1.2 V (Figure 7c). They claimed that overpotential is due to the unsaturated chemical oxygen bond on the SiO_2_ surface, reacting with MXene flakes and building an electric field at the interface of MXene–SiO_2_. The so-fabricated MSC delivered an energy density of 75.5 mWh cm^–3^.^84^

**Figure 8:**
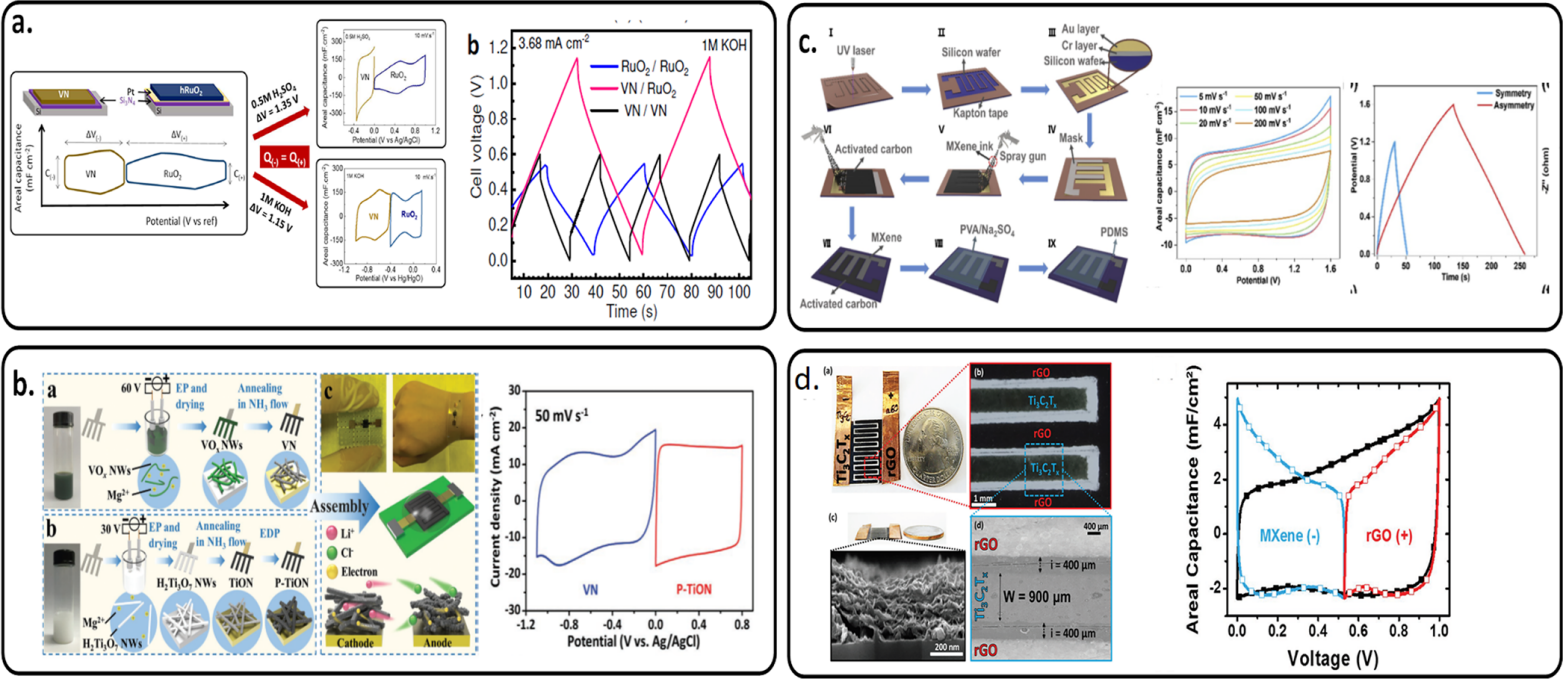
Overview of several asymmetric MSCs on rigid or flexible substrates. (a) Asymmetric VN//RuO_2_ MSC. Reprinted with permission from ref (85). Copyright 2021, Elsevier. (b) Asymmetric VN//TiON MSC. Reprinted with permission from ref (86). Copyright 2020, John Wiley and Sons. (c) Asymmetric Ti_3_C_2_T_*x*_//PPy/MnO_2_ MSC. Reprinted with permission from ref (88). Copyright 2020, Elsevier. (d) Asymmetric Ti_3_C_2_T_*x*_//rGO MSC. Reprinted with permission from ref (89). Copyright 2018, John Wiley and Sons.

In contrast, asymmetric MSCs (hereafter, AMSCs) use two different materials for the two electrodes. Consequently, the cell voltage can be widened (Figure 2b) by appropriately combining two different materials with complementary electrochemical windows potentials. Note that, to reach this goal, a good charge balancing has to be achieved between the two electrodes, by tuning the electrode thicknesses. Benefiting from the complementary working potential window of VN and hydrated RuO_2_ in 1 M KOH electrolyte, Asbani et al. reported AMSC VN//hRuO_2_ with a cell voltage of 1.15 V and an energy density of 20 μWh cm^–2^ at a power density of 3 mW cm^–2^ (Figure 8a). This AMSC was 5 times better when compared to the symmetric VN//VN and hRuO_2_//hRuO_2_ symmetric configuration.^85^ Using VN as the negative electrode but poly(3,4-ethylenedioxythiophene)-coated titanium oxynitride (P-TiON) as the positive electrode, Yang et al. achieved AMSC P-TiON//VN with a cell voltage of 1.8 V in a LiCl/PVA gel electrolyte and an energy density of 32.4 μWh cm^–2^ at a power density of 0.9 mW cm^–2^ (Figure 8b).^86^ Li et al. reported an AMSC Ti_3_C_2_T_*x*_//polypyrrole (PPy)/MnO_2_ that can operate at a cell voltage of 1.2 V in a PVA/H_2_SO_4_ electrolyte, i.e., doubling the 0.6 V cell voltage of a symmetric MSC Ti_3_C_2_T_*x*_ (Figure 8c). This AMSC Ti_3_C_2_T_*x*_//polypyrrole (PPy)/MnO_2_ delivered an energy density of 61.5 mF cm^–2^ at a power density of 6.73 μWh cm^–2^ and a flexible ability inherited from the conducting polymer PPy.^87^ AMSC based on MXene and active carbon (AC) was demonstrated by Xie et al.^88^ This AMSC Ti_3_C_2_T_*x*_//AC delivered a cell voltage of 1.6 V in PVA/Na_2_SO_4_ electrolyte and an energy density of 3.5 mWh cm^–3^ at a power density of 100 mW cm^–3^. Couly et al. reported that AMSC Ti_3_C_2_T_*x*_//rGO (reduced graphene oxide) operated at a cell voltage of 1 V in PVA/H_2_SO_4_ electrolyte and delivered an energy density of 8.6 mWh cm^–3^ at a power density of 0.2 W cm^–3^ and good flexibility due to the use of polyethylene terephthalate (PET) as a substrate (Figure 8d).^89^

## 8 Perspectives and Challenges

In summary, MSCs are efficient miniaturized energy storage devices with high power density, long cycle life, and fast charge/discharge rate, but they suffer from low energy density. Additionally, the technology readiness level (TRL) of such an MSC is low, mainly due to the difficulty fabricating MSCs in a solid-state configuration, i.e., with a solid electrolyte having no solvent evaporation, good ionic conductivity, and a photopatternable capability to limit the footprint surface of the electrolyte to a few square millimeters.

Moving from thin film to thick film is another solution for the fabrication of high performance MSCs since energy density is proportional to the amount of electroactive material. The main challenges deal with the low electrical conductivity of thick films and the ion diffusion within the electrode materials. It is also mandatory to avoid a short circuit between the electrodes. For the first point, it can be solved by employing high conductivity material such as transition metal nitrides such as VN,^31^ W_2_N,^33^ CrN,^111^ and TiN^112^ working as a bifunctional materials (current collector and electrode material). For the second point and specifically with pseudocapacitive film electrodes, an attractive solution consists of “playing” with the nanostructured electrode material as proposed for powder by Dunn et al.^113,114^ and Naoi et al.^115^ Nanosized material allows the active material to be close to the ion coming from the electrolyte solution. Consequently, fast redox reactions occurs in nanostructured pseudocapacitive materials, and the concept is known as extrinsic pseudocapacitance. Following this downsizing engineering concept for electroactive material but taking into account the use of thin film deposition techniques for MSCs, it is interesting to tune the film morphology to produce “porous” pseudocapacitive thin and thick film electrodes combining very high capacitance values with good rate capability. As an example, tuning the deposition pressure during a sputtering process allows modification of the film porosity.^45^ Another approach to tune the film porosity consists of codepositing two elements while chemically etching only one element after the deposition, such as proposed by Pech et al. for 3D MSC based on a gold/copper alloy scaffold.^82^ A last challenge/perspective in the field of MSC consists of the integration of MXene electrode materials in a real device using mass production deposition methods. Advanced microfabrication techniques such as laser scribbling and ion beam or plasma etching processes are exciting solutions to solve the problem of short circuiting during the fabrication process and then to fabricate interdigitated MSCs at the wafer level.

To produce commercially off the shelf MSCs, it is impossible to use liquid electrolytes due to leakage issues. Solid-state ionogel electrolytes are interesting candidates to replace liquid electrolytes because of their unique properties of no water evaporation and photopatternable capabilities^22^ for the microfabrication process. Additionally, a solid-state electrolyte can extend the classical voltage window of liquid electrolytes, hence the performance of MSCs.

At the electrolyte level, another challenge consists of using active redox electrolytes. To improve the performance of an electrochemical capacitor, an attractive solution consists of using biredox ionic liquids to achieve bulk-like redox density at liquid-like fast kinetics. The cation and anion of biredox ionic liquids (IL) can bear moieties that undergo very fast reversible redox reactions: a major demonstration^116^ was achieved by Fontaine et al. in 2016 where BMIM-TFSI ionic liquid was functionalized with anthraquinone (AQ) and 2,2,6,6-tetramethyl-piperidinyl-1-oxyl (TEMPO) moieties. Consequently, the specific capacitance values combine both the charge storage process coming not only from the porous carbon electrodes but also from the AQ and TEMPO moieties within the electrolyte. Based on this proof of concept at the macroscale level for electrochemical capacitors based on porous carbon electrodes, a major challenge in the field of MSC could consist of reproducing these experiments at the micro- or nanoscale level by trapping biredox IL in a confined matrix combined with the ionogel approach taking into account the limited footprint surface of MSCs.

Electrode material and device topology are also key parameters that could be tuned to improve energy density in order to develop the next generation of MSCs. Classical materials such as carbon-based, pseudocapacitive material (metal oxide, metal nitride, or conductive polymer) along with new materials, namely, multicationic or MXene, are attractive candidates at the electrode level to improve the capacitance values. In that context, the key issue remains the deposition of such electrodes as films on a substrate and the ability to pattern/etch the film, taking into account an interdigitated shape. Symmetric and asymmetric configurations are mainly reported for MSCs based on pseudocapacitive materials. AMSCs (asymmetric MSCs) benefit from the complementary working potential window of the two electrode materials to widen the cell voltage of MSCs.

The progress keeps going, and many new strategies can be expected to improve MSCs’ performance. At the material level, the deposition/synthesis of new pseudocapacitive ternary materials (multicationic or multianionic) is an attractive solution to boost the capacitance value/potential windows of an electrode. Nevertheless, stabilizing a phase is the biggest challenge, but it was already demonstrated with various ternary nitrides predicted by Ceder et al.,^117,118^ MnFe_2_O_4_ pseudocapacitive material,^94^ or FeWO_4_.^92^ The pseudocapacitive behavior of MnFe_2_O_4_ or FeWO_4_ is very interesting from a capacitance point of view when tested in an aqueous electrolyte, but the cell voltage of MSC will be limited by the electrochemical window stability of water (1.23 V). An interesting approach for MSCs could be related to the use of electrode materials showing pseudocapacitive properties in an organic electrolyte as demonstrated for bulk electrode by Simon et al. recently with MXene Ti_3_C_2_T_*x*_.^119^ As a consequence, the cell voltage could be increased up to 3 V, but the challenge consists of preparing MXene films using the molten salt synthesis method proposed by the authors.

For the MSC topology, asymmetric interdigitated MSCs have many advantages over other configurations, especially for connecting with microdevices. By combining with the 3D electrode fabrication design,^73^ i.e., where the surface area of active materials is greatly increased, a significant increase in surface capacitance of the electrode is expected for powering the next generation of MSCs.
